# COPD Severity and Five-Year Mortality After Acute Myocardial Infarction in Patients with Chronic Obstructive Pulmonary Disease: A Retrospective Cohort Study

**DOI:** 10.3390/medsci14040486

**Published:** 2026-08-16

**Authors:** Sagi Shashar, Tahel Cohen, Liora Boehm Cohen, Hezzy Shmueli, Arthur Shiyovich, Harel Gilutz, Ygal Plakht

**Affiliations:** 1 Department of Family Medicine, Maccabi Healthcare Services, Tel Aviv, Israel; 2 Department of Nursing, Recanati School for Community Health Professions, Faculty of Health Sciences, Ben-Gurion University of the Negev, Beer Sheva, Israel; taelshana2@gmail.com; 3 Mother and Newborn Department, Barzilai Medical Center, Ashkelon, Israel; 4 Pulmonology Institute, Soroka University Medical Center, Beer Sheva, Israel; lioraboehmcohen@gmail.com; 5 Goldman Medical School, Faculty of Health Sciences, Ben-Gurion University of the Negev, Beer Sheva, Israel; gilutz@bgu.ac.il; 6 Cardiology Division, Hillel Yaffe Medical Center, Hadera, Israel; hezzys@hymc.gov.il; 7 Rappaport Faculty of Medicine, Technion University, Heifa, Israel; 8 Brigham and Women’s Hospital, Heart and Vascular Institute, Mass General Brigham, Boston, MA, USA; arthur.shiyovich@gmail.com; 9 Department of Emergency Medicine, Soroka University Medical Center, Beer-Sheva 84101, Israel

**Keywords:** COPD, acute myocardial infarction, FEV1, spirometry, mortality, survival analysis, GOLD classification

## Abstract

*Background*: Chronic obstructive pulmonary disease (COPD) and acute myocardial infarction (AMI) frequently coexist and share several biological pathways and risk factors. Although COPD is associated with worse outcomes after AMI, the prognostic value of objective pulmonary function has not been well characterized. The aim of this study was to evaluate the association between airflow-obstruction severity and five-year all-cause mortality after AMI among patients with COPD. *Methods*: This single-center retrospective cohort study was restricted to adult post-AMI patients (2002–2017) with a background diagnosis of COPD and documented spirometry; therefore, the final cohort represents a selected subgroup of the source COPD-AMI population. Airflow obstruction was categorized according to forced expiratory volume in one second (FEV1) as Mild, Moderate, Severe, or Very Severe (according to the Global Initiative for Chronic Obstructive Lung Disease (GOLD)). The outcome was all-cause mortality during up to five years of follow-up. *Results*: Among 1594 patients hospitalized with AMI and a COPD diagnosis, 169 patients with available spirometry were included in this selected analytical cohort (mean age 66.76 ± 9.86 years, 76.9% male). During follow-up, 65 patients died, corresponding to an overall five-year mortality rate of 38.5%. Mortality increased progressively across obstruction categories: 19.0%, 33.3%, 40.0%, and 70.8% in Mild, Moderate, Severe, and Very Severe, respectively (*p* for trend <0.001). In multivariable analysis, adjusted hazard ratios for mortality were: 2.49 (95% CI 0.86–7.24), 3.38 (95% CI 1.09–10.46), and 5.23 (95% CI 1.62–16.89) for Moderate, Severe, and Very Severe, compared with Mild obstruction. *Conclusions*: Among patients with COPD who survived hospitalization for AMI, more severe airflow obstruction was associated with substantially higher five-year all-cause mortality. These findings support the potential role of spirometry-based risk stratification and highlight the need for integrated cardiopulmonary follow-up after AMI.

## 1. Introduction

Acute myocardial infarction (AMI) remains a major cause of morbidity and mortality despite substantial improvements in reperfusion strategies, pharmacotherapy and secondary prevention. Long-term outcomes after AMI are influenced not only by the severity of the coronary event, but also by comorbid conditions, functional reserve and the burden of modifiable cardiovascular risk factors [1,2,3,4].

Chronic obstructive pulmonary disease (COPD) is a heterogeneous respiratory condition characterized by persistent respiratory symptoms and airflow limitation related to airway and/or alveolar abnormalities [5,6]. COPD frequently coexists with cardiovascular diseases. This overlap is partly explained by shared risk factors, especially smoking and older age, but also by systemic inflammation, hypoxemia, oxidative stress, endothelial dysfunction, arterial stiffness, and pulmonary vascular disease [7,8,9,10,11,12]. Cardiovascular causes account for a substantial proportion of deaths in COPD, and COPD has repeatedly been associated with worse clinical outcomes following AMI [7,10,13,14].

Forced expiratory volume in one second (FEV1) is central to spirometric assessment for grading of airflow-obstruction severity. Prior work has linked impaired lung function to cardiovascular morbidity, mortality and the complexity of coronary artery disease [15,16,17]. However, evidence is limited regarding whether FEV1-based COPD severity adds prognostic information among patients who have already experienced AMI [18,19]. Spirometry is non-invasive, routinely available and may help identify a subgroup of post-AMI patients requiring closer cardiopulmonary surveillance [20,21].

Therefore, the present study aimed to examine the association between pulmonary function, categorized using FEV1-based GOLD obstruction severity, and five-year all-cause mortality after AMI among patients with COPD. We hypothesized that more severe airflow obstruction would be associated with a graded increase in long-term mortality risk.

## 2. Methods

### 2.1. Study Design and Setting

We conducted a single-center retrospective cohort study based on the Soroka Acute Myocardial Infarction (SAMI) Project, an institutional database of patients hospitalized with AMI at Soroka University Medical Center between 2002 and 2017 [22]. The study used existing hospital data linked to long-term mortality follow-up.

The study was conducted in accordance with the Declaration of Helsinki and approved by the Soroka Medical Center Institutional Review Board (initial approval SOR-0319-16, granted 19 September 2016, extended up to 13 December 2022). Informed consent was waived by the committee, as the study was retrospective and included only anonymized data.

### 2.2. Study Population

The source population included patients aged 18 years or older who were hospitalized with AMI and had a prior diagnosis of COPD. AMI and COPD were identified using International Classification of Diseases, Ninth Revision, Clinical Modification (ICD-9-CM) discharge codes: code 410 for AMI; and codes 491, 492, and 496 for COPD. For patients with more than one AMI hospitalization during the study period, the earliest AMI hospitalization with spirometry available within the prespecified time window was defined as the index hospitalization.

Patients were excluded if they had additional conditions that could materially affect pulmonary function tests, such as asthma, lung malignancy, cardiomyopathy, heart failure, or previous lung surgery; were not Israeli residents; had no documented lung-function test results within one year before or after the index AMI; or died during the index hospitalization.

### 2.3. Exposure Definition

The primary exposure was airflow-obstruction severity based on spirometry performed within one year before or after AMI. Because many pulmonary function test results were available only as scanned reports rather than structured electronic data, spirometry data were manually reviewed and abstracted from patients’ medical records among patients followed in the Soroka Pulmonary Clinic. Patients were categorized according to FEV1 percent predicted using Global Initiative for Chronic Obstructive Lung Disease (GOLD) stages: Mild (FEV1 ≥ 80%), Moderate (50% ≤ FEV1 < 80%), Severe (30% ≤ FEV1 < 50%) and Very Severe (FEV1 < 30%) [23]. FEV1/FVC was also summarized as a continuous lung-function measure.

### 2.4. Outcome

The primary outcome was all-cause mortality during up to five years of follow-up after discharge from the index AMI hospitalization. Mortality data were obtained from linked institutional and national records.

### 2.5. Covariates

Covariates included age, sex, ethnicity, cardiovascular diseases, cardiovascular risk factors, other comorbidities, administrative characteristics of the AMI hospitalization, clinical characteristics of the AMI event, and type of AMI treatment. Disease classifications and interventions were categorized based on ICD-9-CM discharge codes.

### 2.6. Statistical Analysis

Categorical variables are presented as numbers and percentages, and continuous variables as means with standard deviations (SD). Baseline characteristics were compared across obstruction-severity groups using Chi-square tests or Chi-square tests for linear trend for categorical and ordinal variables, and analysis of variance (ANOVA) or ANOVA for linear trend tests for continuous variables. Mortality was compared between groups using Kaplan–Meier analysis and the log-rank test. Crude and adjusted hazard ratios (HR/AdjHRs) with 95% confidence intervals (CIs) were estimated using Cox proportional hazards regression. The Mild obstruction group served as the reference category. The multivariable model included obstruction severity and clinically relevant covariates or variables associated with the outcome. Statistical analyses were performed using IBM SPSS Statistics version 26, and a two-sided *p*-value < 0.05 was considered statistically significant.

### 2.7. Assessment of Selection Related to Spirometry Availability

To assess potential selection related to spirometry availability, baseline characteristics were compared between patients with documented spirometry who were included in the final analytical cohort and eligible COPD-AMI patients without documented spirometry.

In addition, to assess the potential impact of spirometry timing relative to the index AMI, patients were classified according to whether spirometry was performed before or after the index AMI. The distribution of GOLD airflow-obstruction severity was compared between these timing groups, and mortality trends across severity categories were examined separately in patients with pre-AMI and post-AMI spirometry. Because of the limited number of patients with pre-AMI spirometry, a multivariable sensitivity analysis restricted to this subgroup was not performed.

## 3. Results

### 3.1. Study Cohort and Overall Baseline Characteristics

During the study period, 1594 patients were hospitalized with AMI and had a COPD diagnosis. After applying eligibility criteria and excluding patients without documented spirometry or those who died during the index hospitalization, 169 patients were included in the final cohort. Because only patients with available spirometry could be classified according to GOLD airflow-obstruction severity, the final analytical cohort represents a selected subgroup of the source COPD-AMI population. Baseline characteristics of patients with and without documented spirometry are presented in Supplementary Table S1. Patients with available spirometry differed from those without available spirometry in several baseline characteristics, including demographic variables, cardiovascular comorbidities and risk factors, AMI type, and treatment strategy.

Regarding spirometry timing, 52 patients (30.8%) underwent spirometry before the index AMI and 117 patients (69.2%) after the index AMI. The median interval between the AMI admission date and spirometry was 50.2 days (IQR, −46.3 to 143.5; range, −365 to 365), with negative values indicating spirometry performed before AMI. Among patients with pre-AMI spirometry, the median interval was −123.5 days (IQR, −213.6 to −60.8), whereas among patients with post-AMI spirometry, the median interval was 100.9 days (IQR, 32.6 to 196.0). The distribution of GOLD airflow-obstruction severity did not differ significantly between these groups (*p* = 0.352). In both timing groups, mortality increased with worsening airflow-obstruction severity; the *p* for trend was 0.059 among patients with pre-AMI spirometry and 0.002 among patients with post-AMI spirometry.

Most patients included in the final cohort were younger than 65 years and male. Approximately one-third of the participants belonged to Arab or other minority groups. The burden of cardiovascular risk factors was high, especially a history of smoking, dyslipidemia, hypertension, and diabetes mellitus (see Table 1). According to GOLD spirometry airflow-obstruction severity, 12.4% were classified as Mild obstruction, 49.7% as Moderate obstruction, 23.7% as Severe obstruction, and 14.2% as Very Severe obstruction. Mean age was comparable across the four groups; however, the distribution of age categories differed significantly between groups. The proportion of men tended to increase with worsening airflow obstruction, although this difference did not reach statistical significance. Ethnicity was similarly distributed across groups. Most cardiovascular risk factors and cardiac comorbidities were also comparable between groups. In contrast, pulmonary heart disease was significantly more frequent among patients with more severe obstruction.

Characteristics of the index AMI hospitalization also varied with airflow-obstruction severity: STEMI was most frequent in the Moderate group and least frequent in the Very Severe group, while invasive treatment was less commonly performed as obstruction severity increased. Regarding hospitalization characteristics, admission or transfer to the intensive coronary care unit decreased progressively with worsening obstruction, and prolonged length of stay also differed between groups.

As expected, spirometry indices differed markedly across groups, with mean FEV1 decreasing across the groups. A similar graded decline was observed for FEV1/FVC (see Table 1).

### 3.2. Five-Year All-Cause Mortality and Survival Analysis

**Figure 1:**
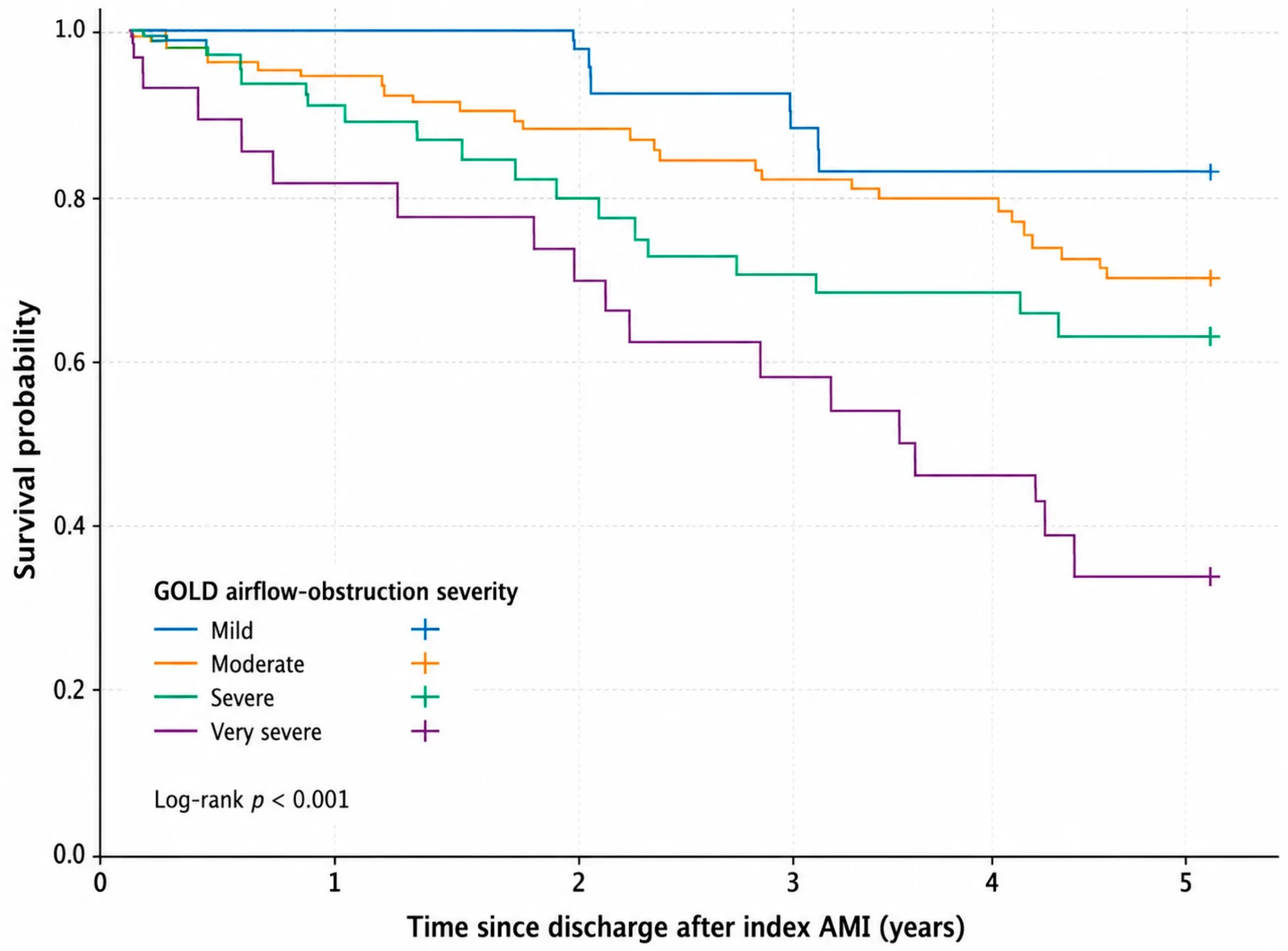
Kaplan–Meier estimates of five-year all-cause survival according to GOLD airflow-obstruction severity. The figure shows Kaplan–Meier survival curves for all-cause mortality during up to five years of follow-up after discharge from the index AMI hospitalization, by GOLD spirometric airflow-obstruction severity. Survival differed significantly across the obstruction-severity groups by the log-rank test (*p* < 0.001). Abbreviations: AMI, acute myocardial infarction; GOLD, Global Initiative for Chronic Obstructive Lung Disease.

Follow-up ranged from 5 days to 5 years, with a median of 5 years and an interquartile range of 3 to 5 years. During this period, 65 of 169 patients died, corresponding to an overall all-cause mortality rate of 38.5%. Cumulative mortality increased progressively according to GOLD airflow-obstruction severity, from 19.0% in the Mild group to 33.3% in the Moderate group, 40.0% in the Severe group, and 70.8% in the Very Severe group (*p* for trend <0.001) (See Figure 1).

### 3.3. Airflow-Obstruction Severity and the Risk for Five-Year All-Cause Mortality

In univariable Cox regression analysis compared with Mild obstruction, the HR for all-cause mortality was 1.90 (95% CI: 0.67–5.42, *p* = 0.229) for Moderate obstruction, 2.53 (95% CI: 0.84–7.56, *p* = 0.098) for Severe obstruction, and 5.54 (95% CI: 1.86–16.50, *p* = 0.002) for Very Severe obstruction. Each one-level increase in obstruction severity was associated with a 1.69-fold higher hazard of five-year mortality. In the multivariable model, the association persisted. Compared with Mild obstruction, AdjHRs were 2.49, 3.38, and 5.23 for Moderate, Severe, and Very Severe obstruction, respectively (see Table 2). Accordingly, for each level increase in obstruction severity, the risk of mortality increased by 1.59 (95% CI: 1.16–2.17, *p* for trend <0.001). Additional variables independently associated with mortality included supraventricular arrhythmias and pulmonary heart disease. Smoking and conservative treatment showed clinically meaningful associations with mortality, although these associations did not reach conventional statistical significance in the adjusted model.

## 4. Discussion

In this retrospective cohort of patients with COPD who survived hospitalization for AMI and had documented spirometry near the index event, the severity of airflow obstruction was strongly and progressively associated with five-year all-cause mortality. Overall mortality was high, affecting more than one-third of the cohort. The mortality gradient was clinically substantial: approximately one in five patients with Mild obstruction died during follow-up, compared with more than two-thirds of patients with Very Severe obstruction. This pattern was also evident in the multivariable model, in which Severe and Very Severe airflow obstruction remained associated with markedly higher mortality risk compared with Mild obstruction. These findings support the study hypothesis and suggest that FEV1-based COPD severity may provide clinically relevant prognostic information after AMI.

Our findings are consistent with the broader literature showing that COPD is not merely a respiratory comorbidity but is considered a systemic condition linked to cardiovascular disease, recurrent events, and adverse outcomes after AMI. Previous systematic reviews and observational studies have shown that COPD is associated with increased risk of MI and higher long-term mortality following MI [7,18,19]. Importantly, much of the earlier literature examined COPD as a binary diagnosis. The present study adds a more granular post-AMI perspective by using objective spirometric severity categories rather than relying only on coded COPD status. This is clinically important because patients with the same administrative COPD diagnosis may have very different physiological reserve and post-MI vulnerability.

The graded association observed in this study is biologically plausible. FEV1 is widely used to grade airflow limitation in COPD, and several population-based and COPD-specific cohorts have linked impaired FEV1 with mortality and cardiovascular morbidity [13,20,21,24]. In the PURE study, reduced FEV1 was an independent predictor of mortality and cardiovascular morbidity across diverse international populations [20]. In the SUMMIT population of patients with moderate COPD and heightened cardiovascular risk, FEV1 was a stronger predictor of all-cause mortality than FVC [21]. Our results extend this concept into a post-AMI COPD cohort, suggesting that worse airflow obstruction may identify a subgroup with reduced cardiopulmonary reserve, higher susceptibility to recurrent ischemia or heart failure, and lower ability to recover after a major coronary event.

Several mechanisms may explain the relationship between severe airflow obstruction and mortality after AMI. COPD is characterized by chronic airway and systemic inflammation; systemic inflammatory markers such as CRP, fibrinogen, and interleukin-6 have been repeatedly linked to COPD severity and cardiovascular risk [25]. Severe COPD may also promote hypoxemia, pulmonary vasoconstriction, pulmonary hypertension, right ventricular strain, endothelial dysfunction, arterial stiffness and oxidative stress [26,27,28]. These mechanisms can aggravate myocardial oxygen supply-demand mismatch, increase arrhythmogenic risk, impair vascular function and limit functional recovery after AMI. The independent association between pulmonary heart disease and mortality in our cohort further supports the relevance of advanced cardiopulmonary involvement as a marker of poor prognosis.

The association between COPD and post-AMI mortality may also be influenced by differences in AMI presentation and treatment. Patients with COPD may present with overlapping respiratory and cardiac symptoms, potentially delaying diagnosis or complicating clinical assessment. Prior studies have also reported that patients with COPD are less likely to receive invasive coronary procedures and some guideline-directed cardiovascular medications after AMI [19,29,30]. In the present cohort, conservative rather than invasive AMI management was associated with higher mortality. Although this association should not be interpreted causally because treatment selection is affected by clinical severity, frailty and comorbidities, it reinforces the need to ensure that COPD alone does not lead to therapeutic nihilism when evidence-based cardiac interventions are otherwise indicated.

The treatment implications are particularly relevant for beta-blockers. Historically, clinicians have often been reluctant to prescribe beta-blockers to patients with COPD because of concerns regarding bronchospasm and worsening lung function. However, more recent evidence indicates that beta-blockers, especially cardio selective agents, are generally safe in patients with COPD who have a cardiovascular indication and may be associated with lower mortality and fewer exacerbations [17]. A systematic review and meta-analysis including both randomized and observational data found that beta-blocker use in patients with COPD and cardiovascular disease was associated with reduced all-cause mortality, while cardio selective beta-blockers were associated with reduced COPD exacerbation risk [17]. Therefore, the post-AMI management of patients with COPD should emphasize guideline-directed cardiac therapy, while individualizing drug selection and monitoring respiratory status.

Another clinical implication is the need for integrated cardiopulmonary follow-up. The present findings suggest that spirometry should not remain an isolated pulmonary test; when available after AMI, it can help identify patients who may benefit from closer surveillance, aggressive secondary prevention, pulmonary treatment optimization, smoking cessation support, vaccination, exercise-based rehabilitation and early recognition of COPD exacerbations. COPD exacerbations themselves have been associated with a subsequent increase in cardiovascular events, particularly during the early period after exacerbation [12]. This reinforces the concept that preventing exacerbations and optimizing COPD management may be part of cardiovascular risk reduction in this population.

The main strength of this study is its use of manually retrieved pulmonary function data in a well-characterized AMI cohort with long-term mortality follow-up. This is an important methodological contribution because spirometry results are often missing from structured hospital datasets and, in this study, many results were available only as scanned PDF reports. The manual retrieval process enabled objective classification of obstruction severity and allowed the study to address a question that is difficult to examine using routine administrative data alone.

## 5. Limitations

Several limitations should be acknowledged. The principal limitation is selection bias related to spirometry availability. Only 169 patients had eligible spirometry data, representing a small fraction of the source COPD-AMI cohort. Although we compared baseline characteristics between patients with and without available spirometry, residual differences that were not captured in the available data may remain. Therefore, the findings should be interpreted as applying primarily to COPD-AMI patients with available spirometric assessment and may not be fully generalizable to all patients with COPD after AMI. In addition, because patients with heart failure or cardiomyopathy were excluded, the findings may not be generalizable to the broader real-world post-AMI COPD population, in whom these conditions are common.

In addition, AMI and COPD diagnoses were based on discharge codes, and post-bronchodilator FEV1/FVC values were not consistently available. Therefore, we could not uniformly confirm that all patients fulfilled the standard spirometric diagnostic criterion for COPD, such as post-bronchodilator FEV1/FVC <0.70. This may have introduced diagnostic misclassification. Moreover, several clinically important variables were unavailable or incompletely captured, including COPD duration, exacerbation history, dyspnea burden, CAT or mMRC scores, oxygen use, inhaled COPD therapy, beta-blocker use, discharge medications, and cause-specific mortality. In addition, we did not compare FEV1-based spirometric severity with other established measures of COPD severity. Therefore, residual confounding cannot be excluded, and the observed associations should be interpreted as prognostic associations rather than causal effects.

In addition, the relatively small number of patients in the Mild and Very Severe groups also resulted in wide confidence intervals, and therefore the magnitude of the hazard ratios should be interpreted cautiously. Furthermore, although mortality increased with worsening airflow-obstruction severity among both patients with pre-AMI and post-AMI spirometry, spirometry performed after AMI may still have been influenced by post-infarction clinical status, heart failure, deconditioning, or treatment changes.

Future studies should validate these findings in larger multicenter cohorts with structured spirometry performed at predefined time points after AMI, ideally including both patients with and without COPD, along with detailed COPD phenotyping, medication data, cardiovascular imaging, and cause-specific outcomes. Further research should also examine whether post-AMI care pathways tailored to COPD severity can improve survival, increase adherence to guideline-directed cardiovascular therapy, reduce COPD exacerbations, and prevent recurrent cardiovascular events. A particularly useful next step would be to compare outcomes among post-AMI COPD patients according to both spirometric severity and treatment intensity, including invasive coronary management, cardioselective beta-blocker use, pulmonary rehabilitation, and optimized inhaled therapy.

## 6. Conclusions

Among patients with COPD and available spirometry who survived hospitalization for AMI, more severe FEV1-based spirometric impairment was associated with substantially higher five-year all-cause mortality. These findings suggest that spirometric severity may provide prognostic information in post-AMI COPD patients and highlight the need for integrated cardiopulmonary follow-up after AMI.

## Figures and Tables

**Table 1:** Baseline characteristics of the study population according to FEV1-based GOLD spirometric severity.

Parameter	Value	GOLD Airflow-Obstruction Severity				Total	*p*
		Mild	Moderate	Severe	Very Severe		
n		21	84	40	24	169	
Demographics							
Age, Years	Mean (SD)	67.52 (11.99)	65.77 (10.62)	67.93 (7.72)	67.58 (8.38)	66.76 (9.86)	0.642
	<65	7 (33.3)	45 (53.6)	10 (25.0)	11 (45.8)	73 (43.2)	0.001
	65–75	7 (33.3)	22 (26.2)	24 (60.0)	9 (37.5)	62 (36.7)	
	≥75	7 (33.3)	17 (20.2)	6 (15.0)	4 (16.7)	34 (20.1)	
Sex	Male	15 (71.4)	60 (71.4)	33 (82.5)	22 (91.7)	130 (76.9)	0.143
Ethnicity	Arab/Other	7 (33.3)	34 (40.5)	12 (30.0)	8 (33.3)	61 (36.1)	0.684
Cardiac diseases							
Supraventricular arrhythmias		3 (14.3)	13 (15.5)	5 (12.5)	3 (12.5)	24 (14.2)	0.967
Pulmonary heart disease		2 (9.5)	6 (7.1)	8 (20.0)	7 (29.2)	23 (13.6)	0.022
CIHD		20 (95.2)	70 (83.3)	35 (87.5)	21 (87.5)	146 (86.4)	0.545
s/p MI		5 (23.8)	11 (13.1)	7 (17.5)	6 (25.0)	29 (17.2)	0.445
Cardiovascular risk factors							
Renal diseases		1 (4.8)	1 (1.2)	3 (7.5)	0	5 (3.0)	0.19
Diabetes mellitus		14 (66.7)	40 (47.6)	22 (55.0)	15 (62.5)	91 (53.8)	0.328
Dyslipidemia		19 (90.5)	63 (75.0)	29 (72.5)	22 (91.7)	133 (78.7)	0.125
Hypertension		14 (66.7)	53 (63.1)	24 (60.0)	16 (66.7)	107 (63.3)	0.938
Smoking		17 (81.0)	68 (81.0)	31 (77.5)	20 (83.3)	136 (80.5)	0.947
PVD		3 (14.3)	10 (11.9)	6 (15.0)	3 (12.5)	22 (13.0)	0.966
Other disorders							
Neurological disorders		7 (33.3)	8 (9.5)	9 (22.5)	0	24 (14.2)	0.003
Malignancy		2 (9.5)	5 (6.0)	2 (5.0)	1 (4.2)	10 (5.9)	0.877
Anemia		9 (42.9)	30 (35.7)	14 (35.0)	7 (29.2)	60 (35.5)	0.82
Administrative characteristics of the hospitalization							
Admitted or transferred to the ICCU		15 (71.4)	55 (65.5)	20(50.0)	8 (33.3)	98 (58.0)	0.015
Length of hospital stay, days	≥7	6 (28.6)	40 (47.6)	26 (65.0)	10 (41.7)	82 (48.5)	0.043
Clinical characteristics of the hospitalization							
Type of AMI	STEMI	5 (23.8)	32 (38.1)	6 (15.0)	2 (8.3)	45 (26.6)	0.006
Treatment strategy	Invasive	17 (81.0)	67 (79.8)	30 (75.0)	12 (50.0)	126 (74.6)	0.026
Spirometry parameters							
FEV1	Mean (SD)	89.33 (9.63)	62.23 (8.20)	40.36 (5.93)	22.46 (5.44)	54.77 (20.91)	<0.001
FEV1/FVC ratio	Mean (SD)	77.44 (7.57)	68.92 (7.68)	60.68 (8.92)	51.21 (16.08)	64.62 (12.49)	<0.001

Data are presented as n (%) for categorical variables and mean ± SD for continuous variables. Patients were stratified into four GOLD spirometric airflow-obstruction severity groups according to FEV1 percent predicted: Mild, Moderate, Severe, and Very Severe obstruction. *p* values represent comparisons across the four obstruction-severity groups. Abbreviations: AMI, acute myocardial infarction; CIHD, chronic ischemic heart disease; FEV1, forced expiratory volume in 1 s; FVC, forced vital capacity; GOLD, Global Initiative for Chronic Obstructive Lung Disease; ICCU, intensive coronary care unit; MI, myocardial infarction; PVD, peripheral vascular disease; SD, standard deviation; STEMI, ST-segment elevation myocardial infarction.

**Table 2:** Multivariable Cox proportional hazards model for five-year all-cause mortality after index AMI.

		Adjusted HR	(95% CI)	*p*
GOLD airflow-obstruction severity	Mild	1 (ref.)		
	Moderate	2.492	(0.858; 7.240)	0.093
	Severe	3.378	(1.091; 10.460)	0.035
	Very Severe	5.225	(1.616; 16.892)	0.006
Age, years	<65	1 (ref.)		
	65–75	0.858	(0.467; 1.577)	0.622
	≥75	1.536	(0.770; 3.061)	0.223
Sex	Male vs. Female	0.723	(0.373; 1.403)	0.338
Smoking		1.918	(0.944; 3.897)	0.072
Prior MI		1.453	(0.790; 2.669)	0.229
Supraventricular arrhythmias		3.072	(1.668; 5.659)	<0.001
Pulmonary heart disease		2.089	(1.084; 4.026)	0.028
Treatment strategy	Invasive vs. conservative	0.605	(0.343; 1.068)	0.083

The table presents adjusted hazard ratios from a multivariable Cox proportional hazards model for all-cause mortality during up to five years of follow-up after discharge from the index AMI hospitalization. The Mild airflow-obstruction group served as the reference category. Hazard ratios greater than 1 indicate a higher hazard of death. Abbreviations: AMI, acute myocardial infarction; CI, confidence interval; HR, hazard ratio; MI, myocardial infarction; ref., reference category.

## Data Availability

The original contributions presented in this study are included in the article/supplementary material. Further inquiries can be directed to the corresponding author.

## Supplementary Materials

The following supporting information can be downloaded at: https://www.mdpi.com/article/10.3390/medsci14040486/s1, Figure S1: Flow diagram of study cohort selection; Table S1. Baseline characteristics of COPD-AMI patients with and without documented spirometry.
