# Supplementary material for: COPD Severity and Five-Year Mortality After Acute Myocardial Infarction in Patients with Chronic Obstructive Pulmonary Disease: A Retrospective Cohort Study
Source: Med Sci (Basel). 2026 Aug 16;14(4):486. doi: 10.3390/medsci14040486 (PMC13515983; doi:10.3390/medsci14040486)
Supplement: Supplementary file 1 [file medsci-14-00486-s001.zip › medsci-4439398-supplementary.pdf]

Supplementary Figure S1. Flow diagram of study cohort selection

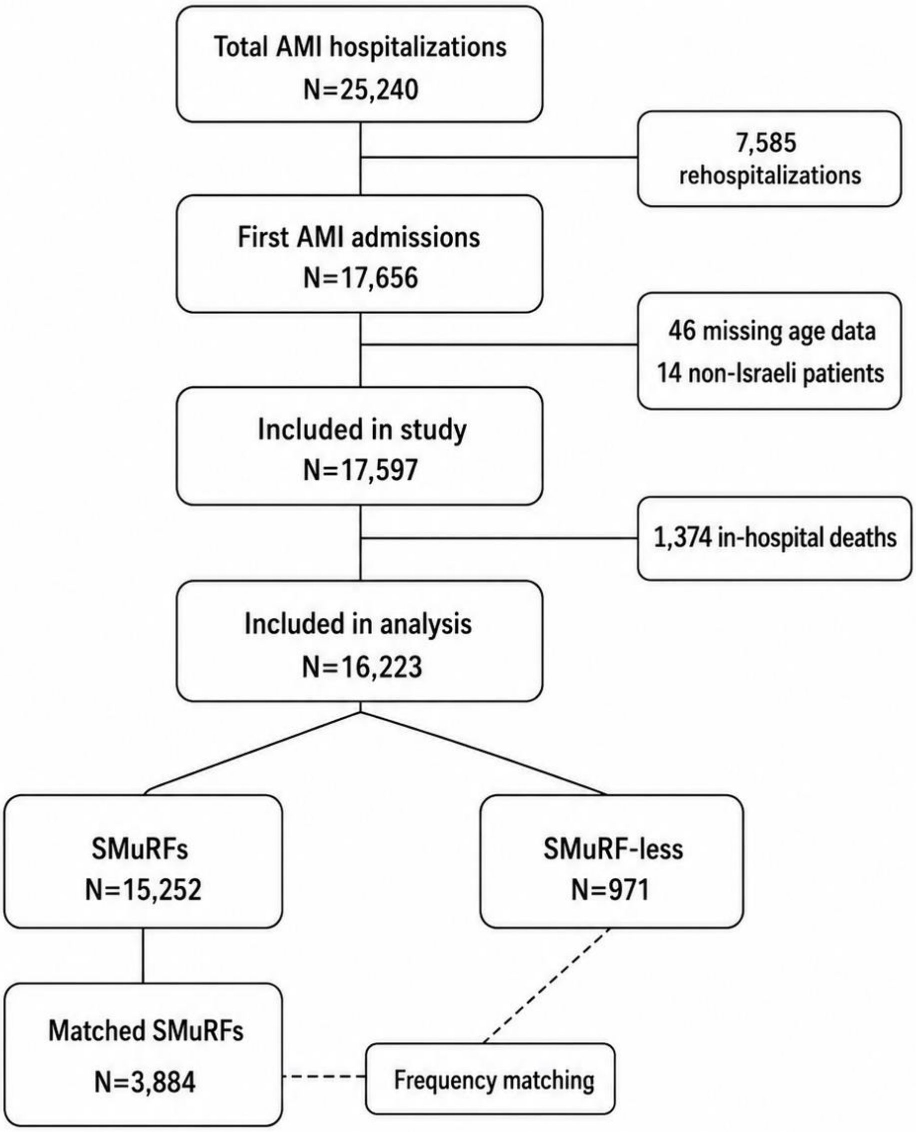

AMI, acute myocardial infarction; SMuRFs, standard modifiable cardiovascular risk factors.

The flow diagram illustrates the derivation of the final analytical cohort from the source population of patients hospitalized with AMI and a documented COPD diagnosis. Patients were excluded because of predefined eligibility criteria, in-hospital mortality during the index hospitalization, or absence of documented spirometry within the predefined time window. The final cohort included 169 patients with available spirometry data.

Supplementary Table S1. Baseline characteristics of COPD-AMI patients with and without documented spirometry

Group		Lung Function Data			P
		No	Yes	Total	
**n**		**767**	**169**	**963**	
**Demographics**					
Age, Years	Mean (SD)	71.91(11.4)	66.76(9.86)		<0.001
	<65	205 (26.7)	73 (43.2)	278 (29.7)	
	65-75	268 (34.9)	62 (36.7)	330 (35.3)	<0.001
	≥75	294 (38.3)	34 (20.1)	328 (35)	
Sex	Male	592 (77.2)	130 (76.9)	722 (77.1)	0.942
Ethnicity	Arab / Other	188 (24.5)	61 (36.1)	249 (26.6)	0.002
**Cardiac diseases**					
Supraventricular arrhythmias		170 (22.2)	24 (14.2)	194 (20.7)	0.021
Pulmonary heart disease		99 (12.9)	23 (13.6)	122 (13)	0.806
CIHD		562 (73.3)	146 (86.4)	708 (75.6)	<0.001
s/p MI		151 (19.7)	29 (17.2)	180 (19.2)	0.45
**Cardiovascular risk factors**					
Renal diseases		74 (9.6)	5 (3)	79 (8.4)	0.005
DM		308 (40.2)	91 (53.8)	399 (42.6)	0.001
Dyslipidemia		589 (76.8)	133 (78.7)	722 (77.1)	0.593
Hypertension		396 (51.6)	107 (63.3)	503 (53.7)	0.006
Smoking		476 (62.1)	136 (80.5)	612 (65.4)	<0.001
**Other disorders**					
Neurological disorders		139 (18.1)	24 (14.2)	163 (17.4)	0.224
Malignancy		36 (4.7)	10 (5.9)	46 (4.9)	0.505
Anemia		385 (50.2)	60 (35.5)	445 (47.5)	<0.001
**Administrative characteristics of the hospitalization**					
Admitted/transposed to ICCU		418 (54.5)	98 (58)	516 (55.1)	0.409
Length of hospital stay, days	≥7	417 (54.4)	82 (48.5)	499 (53.3)	0.168
**Clinical characteristics of the hospitalization**					
Type of AMI	STEMI	278 (36.2)	45 (26.6)	323 (34.5)	0.017
Type of treatment	Invasive	452 (58.9)	126 (74.6)	578 (61.8)	<0.001

Data are presented as n (%) for categorical variables and mean ± standard deviation (SD) for continuous variables. The table compares baseline demographic, clinical, hospitalization-related, and treatment characteristics between COPD-AMI patients included in the final analytical cohort because documented spirometry was available and eligible COPD-AMI patients excluded because spirometry data were unavailable. Abbreviations: AMI, acute myocardial infarction; COPD, chronic obstructive pulmonary disease; SD, standard deviation; CIHD, chronic ischemic heart disease; DM, diabetes mellitus; PVD, peripheral vascular disease; ICCU, intensive coronary care unit; STEMI, ST-segment elevation myocardial infarction.
